# User Authentication Method Based on Keystroke Dynamics and Mouse Dynamics with Scene-Irrelated Features in Hybrid Scenes

**DOI:** 10.3390/s22176627

**Published:** 2022-09-01

**Authors:** Xiujuan Wang, Yutong Shi, Kangfeng Zheng, Yuyang Zhang, Weijie Hong, Siwei Cao

**Affiliations:** 1 Faculty of Information Technology, Beijing University of Technology, Beijing 100124, China; 2 School of Cyberspace Security, Beijing University of Posts and Telecommunications, Beijing 100876, China

**Keywords:** biometrics, keystroke dynamics, mouse dynamics, user authentication

## Abstract

In order to improve user authentication accuracy based on keystroke dynamics and mouse dynamics in hybrid scenes and to consider the user operation changes in different scenes that aggravate user status changes and make it difficult to simulate user behaviors, we present a user authentication method entitled SIURUA. SIURUA uses scene-irrelated features and user-related features for user identification. First, features are extracted based on keystroke data and mouse movement data. Next, scene-irrelated features that have a low correlation with scenes are obtained. Finally, scene-irrelated features are fused with user-related features to ensure the integrity of the features. Experimental results show that the proposed method has the advantage of improving user authentication accuracy in hybrid scenes, with an accuracy of 84% obtained in the experiment.

## 1. Introduction

With the development of computer technology and the internet, increasingly important data and personal information are being stored on computers and on the internet. Therefore, ensuring the security of these data is a growing concern. In recent years, biometric technologies have been widely used. A biometric system is an access control system that can distinguish between legal users and illegal users. Legal users can be authenticated to use the system, while illegal users cannot. Biometric systems allow only legal users to access the system while forbidding access to illegal users, even if they pretend to be legal users. Biometric systems can identify users by the inherent physiological characteristics of the human body (such as fingerprints and the iris) and by behavioral characteristics (such as sound, keystroke habits, and mouse usage habits). Compared with traditional user authentication methods (such as key, username, and password), biometrics has many advantages in that it is difficult to forget, is not easily forged, and is excellent anti-counterfeiting technology. In addition, the successful commercial use of biometrics based on physiological and behavioral characteristics, such as fingerprints, iris, and voice, proves that keystroke dynamics and mouse dynamics have a long-term development prospect.

Biometrics based on keystroke dynamics was first proposed by Gaines et al. in 1980 [1]. Unlike passwords, this method authenticates a user’s identity by the way they type. Keystroke dynamics is an analysis of people’s typing habits, so the key issue is not what the user types but how they type, such as how long they hold down a key or the interval between two keystrokes, which can produce unique patterns and characteristics of an individual. In addition, typing habits are hard to intercept or steal, so keystroke dynamics is an excellent user authentication scheme that can be added to conventional ID/password authentication schemes. Biometrics based on mouse dynamics was first proposed by Ahmed et al. in 2007 [2]. As an analysis of a person’s mouse usage habits, it studied the characteristics of the average moving speed of a mouse in all directions and the time intervals between single clicks or double clicks. The method of combining keystroke dynamics and mouse dynamics was also proposed by Ahmed et al. [3]. Using keystroke and mouse features at the same time enables them to complement each other while ensuring their respective performances so that a better performance can be achieved. Biometric systems based on keystroke dynamics and mouse dynamics can authenticate users when using computers without additional operations and can therefore continue authenticating users after they log into the system. In addition, as both technologies are based on the existing external devices of computers, the cost of authentication methods based on keystroke dynamics and mouse dynamics is lower than for other authentication methods, thus it has stronger generality as well as better development prospects.

In the practical use of computers, users employ the keyboard and mouse for a period of time, and therefore, compared with the fusion of keystroke features and mouse features, detection based only on a single type of feature will reduce the security and stability of the authentication system. Moreover, both keystroke dynamics and mouse dynamics authentication methods are limited to single scenes (such as only focusing on a typing scene) and real scenes (data collected by computers in daily life) [4,5]; however, user authentication in a single scene cannot be applied to real life because the user authentication system cannot determine the computer usage scene, while user authentication in real scenes has low accuracy due to the severe variability of data [6]. Hence, we believe that using multi-scene hybrid data (namely, hybrid scenes), which are close to the real scene data, to train the model can result in the effective authentication of users without accurate scene information. However, as the authentication accuracy of hybrid scenes is lower than that of all single scenes, user authentication accuracy in hybrid scenes is severely reduced.

In order to overcome the above research limitations and to improve the security and stability of user authentication systems, this paper proposes a method based on scene-irrelated features and user-related features that are selected from keystroke dynamics and mouse dynamics features in hybrid scenes. The selected features that have low correlations with scenes are named scene-irrelated features, and those that have high correlations with users are named user-related features. Scene-irrelated features and user-related features are then fused to obtain user-scene features for user authentication. The proposed method is defined as user authentication based on scene-irrelated features and user-related features (SIURUA), and the main contributions of this paper are summarized as follows:

1. A user authentication method is proposed to filter scene-irrelated features in hybrid scenes to reduce the impact of scenes on user authentication;
2. A user authentication method is proposed to fuse scene-irrelated features with user-related features in hybrid scenes.

The remainder of the paper is organized as follows. Section 2 introduces related work. The proposed scene-irrelated features, user-related features, user authentication algorithm, and the evaluation indices of experiments are introduced in Section 3. We provide the experimental configurations and analyses of the experimental results in Section 4. Finally, Section 5 concludes the paper and outlines future work.

## 2. Related Research

### 2.1. Feature Selection

Feature selection is a common method used to improve the accuracy of classification models and can be divided into filter methods, wrapper methods, and embedded methods [7]. Filter methods are independent of machine learning algorithms and use evaluation criteria to enhance the correlations between features and classes and to reduce the correlations between features and features. Wrapper methods use the accuracy of learning algorithms to evaluate feature subsets. Embedded methods automatically select feature subsets in the training process, making up for the shortcomings of filter methods and wrapper methods.

#### 2.1.1. Filter Methods

Filter methods select the features by calculating their weights before using the learning algorithm. The simplest filtering feature selection methods are mutual information [8], chi-square test, and F-test, which all select features based on the correlations between features and labels. In addition, some new filter methods have been proposed recently. Cai et al. [6] proposed a mouse dynamics feature dimension reduction method based on multidimensional scaling (MDS) and isometric feature mapping (ISOMAP). This method generated a weight matrix first, then the ratio of the sum of certain eigenvalues to the sum of total eigenvalues was compared with the threshold. If the ratio was greater than the threshold, the corresponding feature of the eigenvalue was selected as the feature subset. This method could effectively reduce the behavioral variability of mouse dynamics and improve the learning effect.

#### 2.1.2. Wrapper Methods

Wrapper methods select the optimal subset based on an analysis of the pros and cons of feature subsets through models. The most common wrapper method is a feature subset search, such as a genetic algorithm [9] and hill-climbing [10]. Yang et al. proposed a multi-task feature selection method based on a Support Vector Machine (SVM) and a multi-task matrix [11]. This method could optimize the feature weight of a task by using the features of the remaining tasks in multi-task learning. Finally, the feature subset was selected according to the weight to achieve the purpose of improving the learning effect.

#### 2.1.3. Embedded Methods

Embedded methods apply feature selection to the learning process, with the advantage of not needing to evaluate different feature subsets. Common embedded methods include $\ell_{1}$−regularization [12] and $\ell_{2}$−regularization [13]. Regularization is a method of adjusting feature coefficients through the value of a regularization term, and the feature selection is completed when some of the feature coefficients reduce to zero. Feiping et al. [14] proposed a feature selection method based on $\ell_{2,1}$−norm. In a multi-task experiment, the application of $\ell_{2,1}$−norm to the feature coefficient matrix composed of the feature coefficients of each task resulted in the matrix’s rows becoming dense and the columns becoming sparse through learning, so that multi-task feature selection was realized through intertask sharing of information.

### 2.2. Keystroke Dynamics and Mouse Dynamics

#### 2.2.1. Keystroke Dynamics

Since being first proposed by Gaines et al. in 1980 [1], biometric technology based on keystroke dynamics has been developed for 40 years, and some researchers have made considerable progress. Research into keystroke dynamics can be divided into two categories: static authentication based on fixed text and dynamic authentication based on the free text [15].

In recent years, with the development of computer technologies, research into keystroke dynamics has made great advancements. In 2007, Azevedo et al. [16] proposed a hybrid system based on the combination of SVM and genetic algorithm (GA). The experiment obtained a 1.18% False Acceptance Rate (FAR) and 1.58% False Recognition Rate (FRR), while the feature dimension was reduced by 47.51%. Arwa et al. [17] suggested using the fusion method to improve the performance of user authentication based on free text keystroke dynamics. They proposed novel keystroke dynamics features called “semi-timing features”, which had been proved to appear in most users’ keystroke behaviors. The authors combined traditional keystroke dynamics features with “semi-timing features” and used SVM for classification, obtaining 1.56% FAR and 21.5% FRR. Epp et al. [18] applied keystroke dynamics to emotional recognition and achieved an accuracy of between 77% and 80%. Antal et al. [19] fused keystroke dynamics into smartphones and obtained a 15.3% Equal Error Rate (EER). Tsai and Huang [20] put forward a method to detect fraudulent messages through a voting-based statistical classifier to analyze users’ keystroke dynamics, obtaining 10.4% EER. Ayotte et al. [21] proposed a novel instance-based graph comparison algorithm called ITAD that could reduce the keystroke number for user authentication and ultimately obtained 7.8% EER for the Clarkson II dataset and 3.0% EER for the Buffalo dataset. With the development of the Artificial Neural Network (ANN), some neural network-based keystroke dynamics user authentication methods were proposed. For example, Tewari and Verma [22] combined keystroke dynamics data and image data with artificial image data, using AlexNet and ResNet to classify the artificial image, and achieving an accuracy of 98.57%. Lu et al. [23] combined a Convolution Neural Network (CNN) with Recurrent Neural Network (RNN) architecture to test the model using the sliding window extraction of n-gram features, achieving an EER of 2.67% in the best case.

#### 2.2.2. Mouse Dynamics

User authentication based on mouse dynamics can be divided into continuous authentication and static authentication. Continuous authentication means authenticating the user at all times while the user is using the system. Static authentication means authenticating the user under certain circumstances.

Early mouse dynamics research focused on the recognition of user electronic signatures. Higashino et al. [24] used neural networks to study handwriting signatures. In 2003, Everitt et al. [25] conducted a study on signing with a mouse. In 2007, Ahmed et al. [2] verified the feasibility of using mouse movement data to authenticate identity. The proposed features extraction method has been used to date, and the authors obtained 2.46% FAR and 2.46% FRR through experiments. Since then, increasingly more research papers based on mouse dynamics have been published.

Fecher et al. [26] proposed new mouse dynamics features, such as jitters and straightness, and then input these features and other features proposed by Ahmed into a multi-layer classifier based on random forest, finally obtaining 7.5% EER. Kasprowski et al. [27] proposed a biometric method fusing mouse dynamics and eye movement biometrics, finally achieving 92.9% accuracy and 6.8% EER. Gao et al. [28] proposed a continuous authentication method using mouse dynamics based on decision-level fusion. Antal et al. [29] used a convolutional neural network to learn the mouse dynamics features directly, obtaining 0.94 AUC. Hu et al. [30] visualized mouse movements as images and used CNN to classify the images, ultimately attaining 2.94% FAR and 2.28 FRR.

#### 2.2.3. Fusion of Keystroke Dynamics and Mouse Dynamics

A keyboard and a mouse are the main devices by which a user interacts with a computer. Over a period of time, a user will use a keyboard and mouse at the same time to operate a computer, providing the possibility for the fusion of keystroke dynamics and mouse dynamics. Ahmed et al. [3] verified the fusion of keystroke dynamics and mouse dynamics for the first time. They spliced keystroke dynamics and mouse dynamics, then input the features into a neural network, finally obtaining 1.312% FAR and 0.651% FRR. Bailey et al. [31] used the J48 algorithm to achieve decision-level fusion for keystroke dynamics features, mouse dynamics features, and GUI interaction features, finally achieving 2.1% FAR and 2.24% FRR. Mondal et al. [32] proposed a continuous authentication system based on the fusion of keystroke dynamics and mouse dynamics. The confidence of the users of this system depended on the deviation of the users’ operations, with legal users locked after 40,134 operations and illegal users locked after 419 operations. Some studies applied keystroke dynamics and mouse dynamics to soft biometric identification. For example, Earl et al. [33] proposed the use of keystroke and mouse dynamics features to identify users’ gender, handedness, or age, and their research demonstrates that biometric authentication technology can be used in many more areas.

### 2.3. Multiple Kernel Learning

In 1992, Boser et al. [34] introduced the concept of kernel function into machine learning when they researched the support vector machine algorithm. The kernel function maps the linearly inseparable eigenvector x in original feature space to the linearly separable eigenvectors ϕ(x) in high-dimensional space. In fact, choosing the correct kernel has an even stronger impact on the classification results compared with the classifier, but the data may come from different distributions, and it may be necessary to use different kernels for mapping. The objective of multiple kernel learning (MKL) is to combine kernels from multiple sources to improve the accuracy of the target kernel. The parameters used in MKL are generally positive, and the MKL formula is:

(1) $$K=\overset{R}{\underset{r=1}{\sum }}\eta_{r}K_{r},\;\;\;\eta_{r}\geq 0$$

Based on this formula, MKL is used to combine multiple kernels into a large kernel to improve classification accuracy.

The simplest MKL algorithm is AverageMKL, proposed by Belanche et al. [35], in which the parameter of each kernel is the reciprocal of the total number of kernels. Hence, the kernel fusion formula is:

(2) $$k_{\mu }\left(x,z\right)=\overset{P}{\underset{r}{\sum }}\mu_{r}k_{r}\left(x_{r},z_{r}\right),\;\;\;\mu_{r}=\frac{1}{P}\;$$

Kloft et al. [36] and Xu et al. [37] proposed a lasso-based MKL algorithm that uses $\ell_{1}$ −norm to regularize kernel weights. Do et al. [38] found that kernel combination maximizes the decision boundary by fusing the kernel radius into the MKL, naming it Radius MKL (R-MKL). EasyMKL [39] is an improved version of AverageMKL that obtained a combination of kernel parameters through learning.

Multiple kernel learning can improve the accuracy of models by combining multiple types of features. Therefore, MKL has a broader application prospect in machine learning.

## 3. Proposed Approach

**Figure 1:**
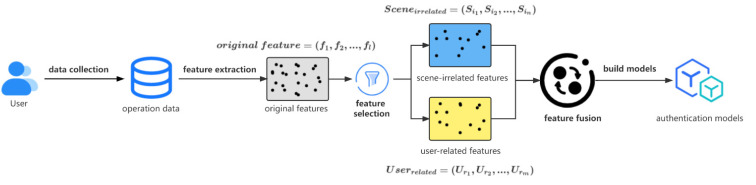
Block diagram of SIURUA.

In this section, we will describe the proposed SIURUA in detail. In order to reduce the impact of different scenes on the hybrid scene features, we propose the selection of scene-irrelated features and user-related features and then propose fusing them to improve the authentication accuracy of the model in hybrid scenes. Figure 1 shows the block diagram of SIURUA. The steps include feature extraction, feature processing, and model training. In Figure 1, we can see the basic process of SIURUA in more detail:

- First, features are extracted from the collected user operation data (details will be provided in Section 3.1);
- Second, scene-irrelated features and user-related features are selected from the original features (details will be elaborated in Section 3.2);
- Finally, scene-irrelated features and user-related features are fused and the model is trained (details will be presented in Section 3.3).

### 3.1. Feature Extraction

The features adopted in this experiment are keystroke dynamics features and mouse dynamics features. We extract features according to different time lengths. Each dimension of the keystroke dynamics feature and mouse dynamics feature is the average of a feature extracted by a user operating within the time length, so the numbers of user operations are varied at different time lengths, and the extracted feature vectors are different. We use the variable time window to represent the length of time used to extract the features; for example, time window = 60 s or time window = 120 s. In the feature extraction section, features are extracted according to the value of the time window, so the value of the time window is the decisive factor determining the amount of information contained in the features.

In the next feature selection section, we select features from the extracted original features based on the user operation, and we define the original features as $Orig-Feature=\left(f_{1},f_{2},\ldots ,f_{l}\right)$, where l is the total number of dimensions of the original features. The original user operation features consist of the directly extracted keystroke dynamics features and the mouse dynamics features. Therefore, we define the keystroke dynamics feature as $Key=\left(k_{1},k_{2},\ldots ,k_{n}\right)$ (as shown in Section 3.1.1) and the mouse dynamics feature as $Mouse=\left(m_{1},m_{2},\ldots ,m_{m}\right)$ (as shown in Section 3.1.2), where n is the dimension size of keystroke dynamics features and m is the dimension size of mouse dynamics features. Then, we directly splice the keystroke dynamics features and mouse dynamics features without additional operations to obtain the user operation original features, as illustrated above. Finally, we obtain the original features $Orig-Feature=\left(f_{1},f_{2},\ldots ,f_{l}\right)=\left(k_{1},k_{2},\ldots ,k_{n},m_{1},m_{2},\ldots ,m_{m}\right)$, where l=n+m. The above features are extracted from the collected keystroke data and mouse data according to the time window, and the values of the time window are 10 s, 20 s, 30 s, 40 s, 50 s, 60 s, 120 s, 180 s, 240 s, 300 s, 360 s, 420 s, and 480 s. In the process of extracting the features, the original features are extracted from all data under the same value of time window, and empty feature components are filled with 0. Finally, we obtain the features shown in Table 1 (i representing the number of features and j representing the dimension of features).

Next, we will elaborate on the extraction methods for keystroke dynamics features and mouse dynamics features.

#### 3.1.1. Keystroke Features

**Figure 2:**
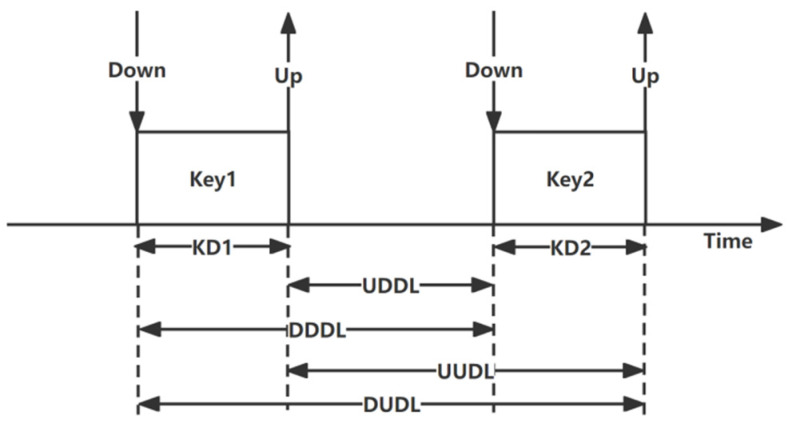
Keystroke dynamics.

We obtain the key pairs according to the time keys are pressed. After the key pairs are obtained, the keystroke features can be extracted [40]. According to the pressing time (down) and the release time (up) of each key, the single key features, and key pair features, as shown in Figure 2, can be obtained (in milliseconds) [41]:

1. Single key features: keystroke duration (KD). Each key pair has two single key features:
  - the keystroke duration of the first key (KD1);
  - the keystroke duration of the second key (KD2).
2. Key pair features: the diagram latency between two keys. Each key pair has six key pair features:
  - down-down diagram latency (DDDL);
  - down-up diagram latency (DUDL);
  - up-down diagram latency (UDDL);
  - up-up diagram latency (UUDL).

The above keystroke features can be applied to every key and every key pair. For a full-size keyboard with 110 keys, the KD features of a single key are 110 dimensions, and the DDDL, DUDL, UDDL, and UUDL features of the key pairs are 12,100 dimensions; thus, there are 48,400 dimensions in total. Hence, the keystroke features in each time window have 48,510 dimensions. Therefore, the keystroke dynamics feature dimension n (defined in Section 3.1) is equal to 48,510, and the keystroke dynamics features are expressed as $Key=\left(k_{1},k_{2},\ldots ,k_{48510}\right)$.

#### 3.1.2. Mouse Features

**Figure 3:**
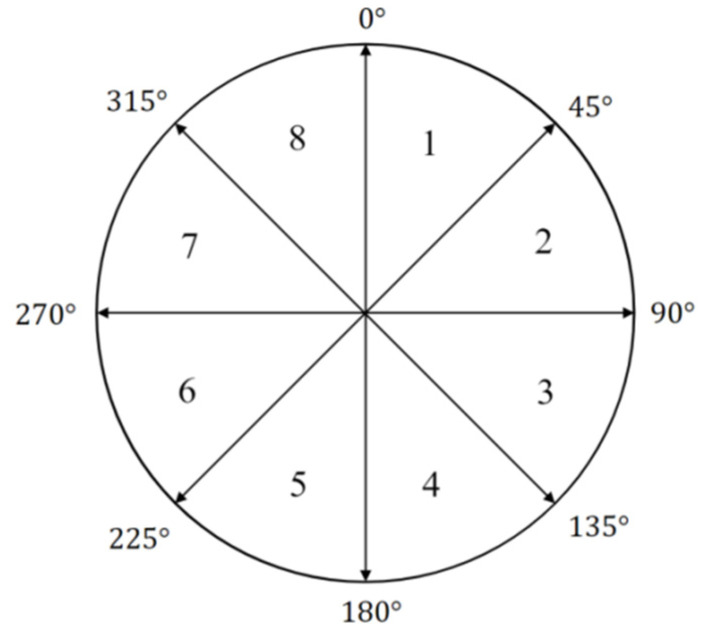
Eight directions for mouse dynamics feature extraction.

We extract mouse dynamics features according to the operation types [2]. On the basis of the recorded mouse movement data—left-click data, right-click data, and eight movement directions are shown in Figure 3—we can extract seven types of mouse features:

- **Movement speed compared with traveled distance (MSD):** The average speed of all moves within different moving distances. The range of moving distance is every interval of 100 pixels within 1–800 pixels (such as 1–100 pixels, 101–200 pixels). The length of the MSD feature vector is 8;
- **Average movement speed per movement direction (MDA):** The average speed of all movements in different moving directions. The moving direction is divided into eight equal parts. The length of the MDA feature vector is 8;
- **Average speed for different types of actions (ATA):** The average mouse movement speed of different operation types. There are three operations, namely, mouse-move, drag-drop, and point-click. The length of the ATA feature vector is 3;
- **Traveled distance histogram (TDH):** The ratio of the number of mouse operations in different moving distances to the total number of mouse operations. The length of the TDH feature vector is 8;
- **Movement direction histogram (MDH):** The ratio of the number of mouse operations in different moving directions to the total number of mouse operations. The length of the MDH feature vector is 8;
- **Actions types histogram (ATH):** The ratio of the number of mouse operations of different operation types to the total number of mouse operations. The length of the ATH feature vector is 3;
- **Movement elapsed time histogram (MTH):** The ratio of the number of mouse operations in different operation durations to the total number of mouse operations. The time range is 5 time periods separated by 200 milliseconds within 1–1000 milliseconds. The length of the MTH feature vector is 5.

We combine the above features into a feature vector, finally obtaining 43-dimensional mouse dynamics features. Therefore, the mouse dynamics feature dimension m (defined in Section 3.1) is equal to 43, and the mouse dynamics features are expressed as $Mouse=\left(m_{1},m_{2},\ldots ,m_{43}\right)$.

### 3.2. Feature Processing

#### 3.2.1. Scene-Irrelated Features and User-Related Features

As single scene data are collected in restricted environments, we consider that there will be some changes in users’ keystrokes and mouse operations in different scenes, and we call the factors that lead to those changes “scene information”. Due to the scene information, we consider that features that are highly correlated with scenes will affect user authentication accuracy. Therefore, scene-irrelated features can be selected to effectively distinguish users and reduce the correlation between features and scenes. We calculate the correlation $COR_{scene}\left(f_{i}\right)$ between each dimension of features $f_{i}$ and scenes. Based on these data, we obtain a sequence of scene correlation degree:

(3) $$Scene-COR=\{COR_{scene}\left(f_{1}\right),\;COR_{scene}\left(f_{2}\right),\;\ldots ,COR_{scene}\left(f_{j}\right)\},$$

and then select n dimensions of features according to the inequality:

(4) $$COR_{scene}\left(f_{i}\right)<=COR_{scene}\left(f_{n}\right),$$

where $COR_{scene}\left(f_{n}\right)$ is the n-th lowest correlation and $COR_{scene}\left(f_{i}\right)$ is any correlation less than or equal to $COR_{scene}\left(f_{n}\right)$ in Scene−COR. Finally, we obtain n-dimensional features that have the lowest correlation with scenes. We name them “scene-irrelated features” and define them as $Scene_{irrelated}=\left(S_{i_{1}},\;S_{i_{2}},\;\ldots ,S_{i_{n}}\right)$.

Contrary to the scene-irrelated features, some features will have great differences between different users and will have few differences for the same user. These features are more distinguishable to users than other features, therefore we call these features “user-related features”. Using user-related features to classify can achieve excellent results. We calculate the correlation $COR_{user}\left(f_{i}\right)$ between each dimension of features $f_{i}$ and users. Based on these data, we obtain a sequence of user correlation degree:

(5) $$\mathrm{User}-\mathrm{COR}=\{COR_{user}\left(f_{1}\right),\;COR_{user}\left(f_{2}\right),\;\ldots ,COR_{user}\left(f_{j}\right)\},$$

and then select m dimensions of features according to the inequality:

(6) $$COR_{user}\left(f_{i}\right)>=COR_{user}\left(f_{m}\right),$$

where $COR_{user}\left(f_{m}\right)$ is the m-th lowest correlation and $COR_{user}\left(f_{i}\right)$ is either correlation in User−COR. Finally, we obtain m-dimensional features that have the highest correlation with users. We name them as user-related features and define them as $User_{related}=\left(U_{r_{1}},\;U_{r_{2}},\;\ldots ,U_{r_{m}}\right)$.

We consider that user-related features still contain some scene information in hybrid scenes; the ratio of scene information contained in user-related features can be reduced by fusing scene-irrelated features (we call the generated features “user-scene features”), which can improve the user authentication accuracy in hybrid scenes. We define user-scene features as $User-Scene=K\left(U_{r},S_{i}\right)$, where K(·) is the kernel fusion function (which will be introduced in Section 3.3).

#### 3.2.2. Feature Selection Method

The SIURUA algorithm searches for scene-irrelated features and user-related features, so it is necessary to measure the correlations between features and users and the correlations between features and scenes. As mutual information cannot effectively reflect the correlation between two datasets when the features have many values, adjusted mutual information (AMI) is used to calculate the user correlation and scene correlation of our SIURUA algorithm [42]. First, we number the user sequence $User=\left(u_{1},u_{2},\ldots ,u_{i}\right)$ according to 41 users, and the scene sequence $Scene=\left(s_{1},s_{2},\ldots ,s_{i}\right)$ according to 4 scenes, and separate each dimension of the original features Orig−Feature to obtain the sequence $f_{j}=\left(f_{1,j},f_{2,j},\ldots ,f_{i,j}\right)$. We subsequently calculate $COR_{user}\left(f_{j}\right)=AMI\left(f_{j},User\right)$ and $COR_{scene}\left(f_{j}\right)=AMI\left(f_{j},Scene\right)$, where $AMI\left(f_{j},User\right)$ and $AMI\left(f_{j},Scene\right)$ are calculated using the AMI calculation formula introduced in [42]:

(7) $$AMI\left(f_{j},User\right)=\frac{MI\left(f_{j},User\right)-E\left\{MI\left(f_{j},User\right)\right\}}{avg\left\{H\left(f_{j}\right),H\left(User\right)\right\}-E\left\{MI\left(f_{j},User\right)\right\}}$$

and

(8) $$AMI\left(f_{j},Scene\right)=\frac{MI\left(f_{j},Scene\right)-E\left\{MI\left(f_{j},Scene\right)\right\}}{avg\left\{H\left(f_{j}\right),H\left(Scene\right)\right\}-E\left\{MI\left(f_{j},Scene\right)\right\}},$$

where $E\left\{MI\left(f_{j},User\right)\right\}$ and $E\left\{MI\left(f_{j},Scene\right)\right\}$ represent the expectations of mutual information $MI\left(f_{j},User\right)$ and $MI\left(f_{j},Scene\right)$; and $H\left(f_{j}\right)$, H(User), and H(Scene) are the entropies of $f_{j}=\left(f_{1,j},f_{2,j},\ldots ,f_{i,j}\right)$, $User=\left(u_{1},\ldots \ldots ,u_{i}\right)$, and $Scene=\left(s_{1},s_{2},\ldots ,s_{i}\right)$, respectively.

The calculation formulas of the entropies mentioned above are $H\left(f_{j}\right)=-\overset{i}{\underset{m=1}{\sum }}P\left(f_{m,j}\right)logP\left(f_{m,j}\right)$, $H\left(User\right)=-\overset{i}{\underset{m=1}{\sum }}P\left(u_{m}\right)logP\left(u_{m}\right),$ and $H\left(Scene\right)=-\overset{i}{\underset{m=1}{\sum }}P\left(s_{m}\right)logP\left(s_{m}\right)$, where log denotes the logarithm with a base of two. P(·) is the occurrence with a probability of $f_{m,j}$, $u_{m}$, and $s_{m}$, where 1≤m≤i.

In addition, the expectation of mutual information is introduced in [42], and therefore $MI\left(f_{j},User\right)$ and $MI\left(f_{j},Scene\right)$ are calculated as follows:

(9) $$\begin{array}{cc} E\left\{MI\left(f_{j},User\right)\right\} & =\overset{M_{j}}{\underset{k=1}{\sum }}\overset{41}{\underset{l=1}{\sum }}\overset{min\left(a_{k,j},b_{l}\right)}{\underset{nu_{kl=max\left(a_{k,j}+b_{l}-N_{U},0\right)}}{\sum }}\frac{nu_{kl}}{N_{U}}log\left(\frac{N_{U}\cdot nu_{kl}}{a_{k,j}b_{l}}\right)\\ & \times \frac{a_{k,j}!b_{l}!\left(N_{U}-a_{k,j}\right)!\left(N_{U}-b_{l}\right)!}{N_{U}!nu_{kl}!\left(a_{k,j}-nu_{kl}\right)!\left(b_{l}-nu_{kl}\right)!\left(N_{U}-a_{k,j}-b_{l}+nu_{kl}\right)!} \end{array}$$

and

(10) $$\begin{array}{cc} E\left\{MI\left(f_{j},Scene\right)\right\} & =\overset{M_{j}}{\underset{k=1}{\sum }}\overset{4}{\underset{l=1}{\sum }}\overset{min\left(a_{k,j},c_{l}\right)}{\underset{ns_{kl=max\left(a_{k,j}+c_{l}-N_{S},0\right)}}{\sum }}\frac{ns_{kl}}{N_{S}}log\left(\frac{N_{S}\cdot ns_{kl}}{a_{k,j}c_{l}}\right)\\ & \times \frac{a_{k,j}!c_{l}!\left(N_{S}-a_{k,j}\right)!\left(N_{S}-c_{l}\right)!}{N_{S}!ns_{kl}!\left(a_{k,j}-ns_{kl}\right)!\left(c_{l}-ns_{kl}\right)!\left(N_{S}-a_{k,j}-c_{l}+ns_{kl}\right)!} \end{array}$$

where $M_{j}$ is the number of clusters of clustering $f_{j}=\left\{F_{1,j},\;F_{2,j},\;\ldots ,\;F_{M_{j},j}\right\}$. Similarly, the number of clusters of clustering $User=\left\{U_{1},\;U_{2},\ldots ,\;U_{41}\right\}$ and $Scene=\left\{S_{1},\;S_{2},S_{3},S_{4}\right\}$ are 41 and 4, respectively. In addition, $a_{k,j}=\left|F_{k,j}\right|$, $b_{l}=\left|U_{l}\right|$, $c_{l}=\left|S_{l}\right|$, $nu_{kl}=\left|F_{k,j}\cap U_{l}\right|,$ and $ns_{kl}=\left|F_{k,j}\cap S_{l}\right|$. Finally, $N_{U}=\underset{kl}{\sum }\left|F_{k,j}\cap U_{l}\right|$ and $N_{S}=\underset{kl}{\sum }\left|F_{k,j}\cap S_{l}\right|$.

For example, we have a set of data, as shown in Table 2, which is a feature component, and each dimension of features has its corresponding Scene label.

First, calculate the information entropy and mutual information of $f_{1}$ and Scene to obtain $H\left(f_{1}\right)=1.33218$ and H(Scene)=1.05492, so $avg\left\{H\left(f_{1}\right),H\left(Scene\right)\right\}=1.19355$. Second, calculate the mutual information and the expectation of mutual information of $f_{1}$ and Scene to obtain $MI\left(f_{1},Scene\right)=1.05492$ and $E\left\{MI\left(f_{1},Scene\right)\right\}=0.83311$. Finally, calculate the adjusted mutual information $AMI\left(f_{1},Scene\right)=0.61539$.

The relationships between these variables are shown in Table 3 and Table 4.

After obtaining the $AMI\left(f_{j},User\right)$ and $AMI\left(f_{j},Scene\right)$ of each feature dimension, all values are classified into two sequences of correlation degree: sequence of user correlation degree $\mathrm{User}-\mathrm{COR}=\{COR_{user}\left(f_{1}\right),\;COR_{user}\left(f_{2}\right),\;\ldots ,COR_{user}\left(f_{48553}\right)\}=\left\{AMI\left(f_{1},User\right),AMI\left(f_{2},User\right),\ldots ,AMI\left(f_{48553},User\right)\right\}$ and sequence of scene correlation degree $Scene-COR=\{COR_{scene}\left(f_{1}\right),\;COR_{scene}\left(f_{2}\right),\;\ldots ,COR_{scene}\left(f_{48553}\right)\}=\left\{AMI\left(f_{1},Scene\right),AMI\left(f_{2},Scene\right),\ldots ,AMI\left(f_{48553},Scene\right)\right\}$. The values of User−COR are sorted from largest to smallest, and the values of Scene−COR are sorted from smallest to largest, then the user-related features $User_{related}$ and scene-irrelated features $Scene_{irrelated}$ are selected as the features used for the authentication model.

### 3.3. Feature Fusion Based on MKL

The scene-irrelated features and user-related features that are obtained by the method described in Section 3.2 can improve authentication accuracy. The authentication accuracy can be further improved by fusion. As the two sets of features are composed of different feature components, the two sets of features have different contributions to user authentication. Therefore, feature fusion is essential for combining the advantages of the two groups of features to improve the user authentication accuracy of SIURUA. In this process, EasyMKL is adopted to fuse scene-irrelated features and user-related features. Support vector machine is used for classification. EasyMKL is an improved multiple kernel learning algorithm compared with other multiple kernel learning algorithms represented by AverageMKL. EasyMKL can obtain the optimal parameter combination through learning, and different parameters can provide different weights to each kernel function. This characteristic of EasyMKL is applicable to the proposed fusion of scene-irrelated features and user-related features, as user-related features are helpful in classifying users, and scene-irrelated features can contribute to diluting the scene information in user-related features while classifying users. The two features have different contributions to user authentication, so EasyMKL is chosen for kernel fusion to ensure that the two features can obtain the optimal weight.

EasyMKL can be used to fuse scene-irrelated features and user-related features through two kinds of kernel changes and weights and then combine them for classification. For example, the RBF kernel is used for the scene-irrelated features, and the linear kernel is used for the user-related features. Here is an example of the formula, and kernel function selection is described in detail in Section 4.2.4. In this case, the corresponding multiple kernel learning formula proposed in [39] becomes:

(11) $$K\left(Scene_{irrelated},User_{related}\right)=\mu_{1}k_{1}\left(S_{i_{i}},S_{i_{j}}\right)+\mu_{2}k_{2}\left(U_{r_{i}},U_{r_{j}}\right),\;\;\mu_{1}\geq 0\wedge \mu_{2}\geq 0\wedge \;\mu_{1}+\mu_{2}=1$$

where $k_{1}\left(S_{i_{i}},S_{i_{j}}\right)=exp\left(-\frac{{\left|\left|S_{i_{i}}-S_{i_{j}}\right|\right|}_{1}}{\sigma^{2}}\right)$ is the RBF kernel, and $k_{2}\left(U_{r_{i}},U_{r_{j}}\right)=U_{r_{i}}{}^{T}U_{r_{j}}$ represents the linear kernels, $S_{i_{i}}$, $S_{i_{j}}$, $U_{r_{i}}$, and $U_{r_{j}}$, which, as described in Section 3.2, are the components of $Scene_{irrelated}$ and $User_{related}$. $\mu_{1}$ and $\mu_{2}$ are the weights of $k_{1}\left(\cdot \right)$ and $k_{2}\left(\cdot \right)$. Taking Formula (11) as an example, the User−Scene features are obtained.

## 4. Experiments and Result Analysis

This section aims to validate the feasibility of SIURUA through a series of experiments and compare it with existing algorithms to analyze the experiment results.

### 4.1. Experiment

#### 4.1.1. Data Collection

Forty-five students from the Beijing University of Technology participated in the data collection work. They were asked to perform four tasks (typing, Taobao, Weibo, and gaming) on the same laptop, with each task taking one hour. User data of those that did not complete all four tasks were filtered. Finally, the data of 41 users remained; that is, the total number of users in the experiment is N=41.

The four tasks involved are the most common ones performed by users of computers. Typing required users to type the same article on Microsoft Office Word; Taobao asked users to browse Taobao; in the Weibo data collection process, users browsed Weibo; and in the gaming data collection process, users played a home-made game. Among the four tasks, the typing task was mainly used to collect keystroke data, the gaming task mainly collected mouse movement data, and the remaining two tasks mainly required a mouse to operate, so we were able to collect a large amount of mouse movement data and a small amount of keystroke data.

The home-made gaming task tested the user’s ability to control the mouse. The game randomly displayed circles on the screen, with the circles gradually shrinking until they disappeared. The users needed to click on the circle before the circles disappeared. If users missed any circles, the game would end. The data collection program collected the user’s keyboard and mouse operation details. The keyboard data recorded the pressing and releasing time for each key, and the mouse data recorded the mouse movement, click, release, and scroll times, along with the coordinates on the screen. The collected data were saved in text files.

**Figure 4:**
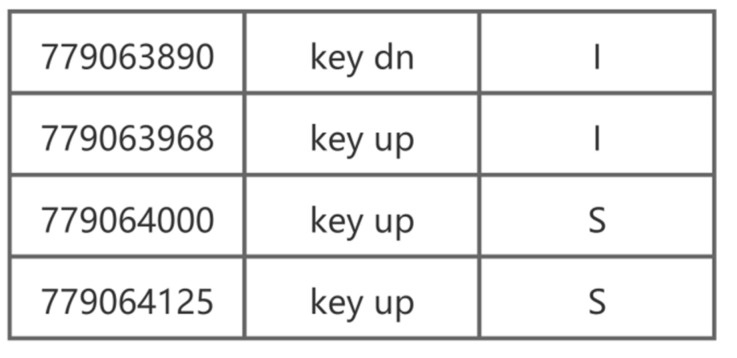
Example of collected keystroke data.

**Figure 5:**
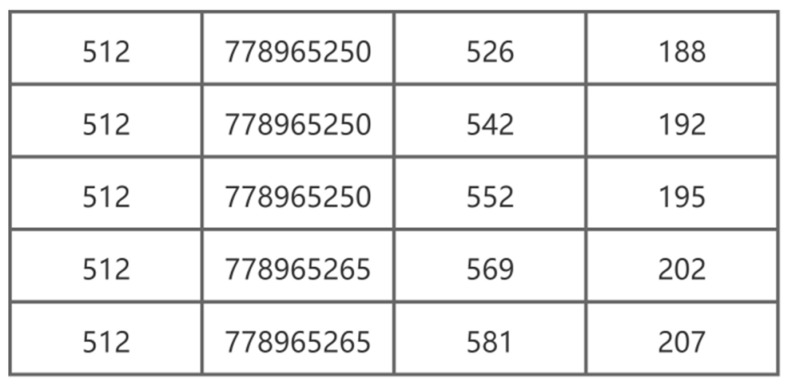
Example of collected mouse data.

The final collected data format samples are shown in Figure 4 and Figure 5. Figure 4 shows an example of the collected keystroke data. The first column represents the time in milliseconds; in the second column, “key dn” means that the key is pressed and “key up” means that the key is released, and the third column shows the data regarding which keys were recorded. Therefore, Figure 4 corresponds to the process of the user inputting the word “IS”. Figure 5 shows a sample of mouse movement data. The first column represents the type of mouse operation (e.g., 512 represents mouse movement), the second column shows the operation time in milliseconds, the third column displays the x-axis coordinate of the screen position of the mouse, and the fourth column represents the mouse y-axis coordinates.

The equipment used in the data collection process of this experiment is Apple MC968CH/A, the processor of this equipment is Intel i5-2257M@1.70 GHz, the memory size is 4.00 GB, and the system is 64-bit Windows 10 Professional Edition.

#### 4.1.2. Process Description

After obtaining keystroke data and mouse movement data, we extracted keystroke dynamics features and mouse dynamics features from the collected data, with the feature extraction performed within a time window. If there was more than one same operation in a time window, the average operation value was calculated as the feature value (as described in Section 3.1). The following are the detailed steps of the experiment process:

1. If user  i (1 ≤ i ≤ N)  is marked as a legal user, the remaining 40 (N−1) users are marked as illegal users. The legal user data are taken as positive samples, and the illegal user data are taken as negative samples;
2. In order to prevent data imbalance, negative samples are under-sampled, and the same number of negative samples as positive samples are randomly obtained;
3. After combining the positive and negative samples, the scene-irrelated features and user-related features are selected and fused (according to the method described in Section 3.2) to obtain the user-scene features User−Scene used for classification;
4. The classification models based on a support vector machine and MKL are trained and tested by 5-fold cross-validation using the obtained User−Scene features.

Steps 1–4 are repeated 41 times until each user acts as a positive sample, and 41 models are trained and tested with each user. Finally, the experiment results of 41 users are averaged to evaluate the performance of SIURUA.

#### 4.1.3. Evaluation

After obtaining the authentication model of a user through the method described in Section 4.1.2, we can calculate the indicators of true positive (TP), false positive (FP), true negative (TN), and false negative (FN). After obtaining the above four basic indicators, we can calculate the well-known evaluation indicators in this field, including accuracy, precision, true positive rate (TPR), false positive rate (FPR), false accept rate (FAR), and false reject rate (FRR), and the F1 scores are used to estimate the classification quality of the model. The calculation methods for FPR and FAR are the same, so they have the same evaluation index. We calculate the above indicators of the model of user i through Formulas (12)–(17):

(12) $$Acc_{i}=\frac{TP_{i}+TN_{i}}{TP_{i}+TN_{i}+FP_{i}+FN_{i}}$$

(13) $$Prec_{i}=\frac{TP_{i}}{TP_{i}+FP_{i}}$$

(14) $$TPR_{i}=\frac{TP_{i}}{TP_{i}+FN_{i}}$$

(15) $$FAR_{i}\left(FPR_{i}\right)=\frac{FP_{i}}{FP_{i}+TN_{i}}$$

(16) $$FRR_{i}=\frac{FN_{i}}{FN_{i}+TP_{i}\;}$$

(17) $$F1-Score_{i}=\frac{2TP_{i}}{2TP_{i}+FP_{i}+FN_{i}}$$

Since this experiment trains a model for each user, the average of the above evaluation indices is calculated in the experiment to evaluate the classification quality. We named these evaluation indices: average accuracy (aAcc), average precision (aPrec), average true positive rate (aTPR), average false positive rate (aFPR), average false accept rate (aFAR), average false reject rate (aFRR), and average F1 score (aF1), and their definitions are shown in Formulas (18)–(23):

(18) $$aAcc=\frac{\sum_{i=1}^{N}Acc_{i}}{N}$$

(19) $$aPrec=\frac{\sum_{i=1}^{N}Prec_{i}}{N}$$

(20) $$aTPR=\frac{\sum_{i=1}^{N}TPR_{i}}{N}$$

(21) $$aFAR\left(aFPR\right)=\frac{\sum_{i=1}^{N}FAR_{i}}{N}$$

(22) $$aFRR=\frac{\sum_{i=1}^{N}FRR_{i}}{N}$$

(23) $$aF1=\frac{\sum_{i=1}^{N}F1-Score_{i}}{N}$$

### 4.2. Analysis of Experimental Results

#### 4.2.1. Illustrate the Reduction in User Authentication Accuracy of Hybrid Scenes

**Figure 6:**
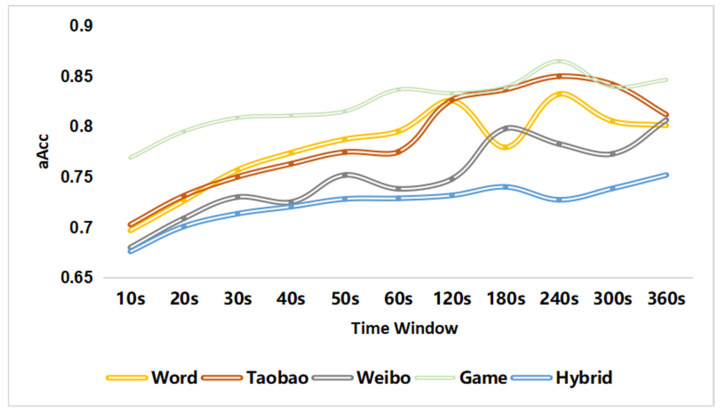
Comparing the user authentication accuracy of hybrid scenes and single scenes.

To verify the viewpoint that “the authentication accuracy of a hybrid scene is lower than that of all single scenes”, we trained the user authentication model based on a support vector machine with a linear kernel in a single scene and a hybrid scene consisting of four scenes. Figure 6 provides the aAcc of the authentication models. It can be seen that in each time window, the authentication aAcc of the hybrid scenes is lower than that of the signal scenes. Therefore, improving the accuracy of user authentication in hybrid scenes is valuable.

#### 4.2.2. Determine Suitable Feature Combination

**Figure 7:**
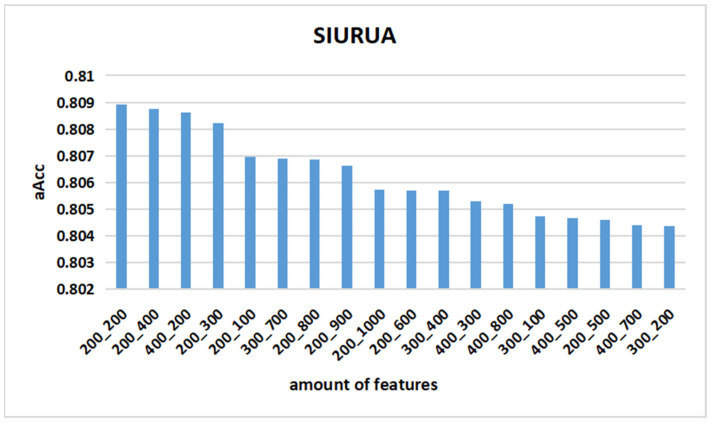
Accuracy of SIURUA in each feature combination.

In the process of using machine learning algorithms to classify, the number of selected features has an impact on the classification quality. This research has two steps for feature selection: selecting the scene-irrelated features and selecting the user-related features. The selected feature number of these two feature selection processes will affect the classification quality; therefore, this experiment is to determine the best-selected feature number. Figure 7 shows 18 feature combinations; for example, 300_200 represents the combination of 300 user-related features and 200 scene-irrelated features. It can be seen that the optimal aAcc (80.86%) is obtained when the features consist of 200 user-related features and 200 scene-irrelated features. Therefore, the suitable feature combination chosen for the SIURUA algorithm combines 200 user-related features and 200 scene-irrelated features.

#### 4.2.3. Determining Suitable Time window

**Figure 8:**
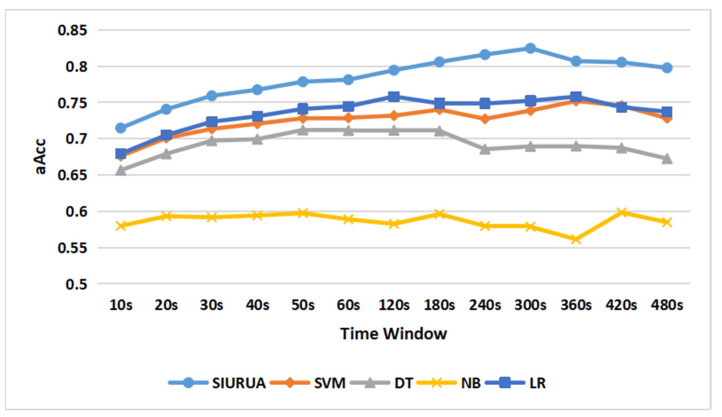
The accuracy of each algorithm in different value of time windows.

The different values of the time window selected during feature extraction will have a certain impact on the classification algorithm; therefore, this experiment is introduced to determine a suitable time window. Specifically, the time windows selected in this experiment are 10 s, 20 s, 30 s, 40 s, 50 s, 60 s, 120 s, 180 s, 240 s, 300 s, 360 s, 420 s, and 480 s. We combined 200 user-related features and 200 scene-irrelated features based on the experience described in Section 4.2.2. As shown in Figure 8, we used SIURUA, SVM with linear kernel, decision tree (DT), logistic regression (LR), and naive Bayes (NB) to classify the hybrid scene data. SIURUA uses a combination of 200 user-related features and 200 scenario-irrelated features, while the other algorithms use all features. It can be seen from Figure 8 that SIURUA achieved the maximum aAcc (82.5%) at 300 s. SVM and LR achieved the maximum aAcc (75.2% and 75.8%, respectively) at 360 s. DT gained the maximum aAcc (71.2%) at 120 s. NB achieved the maximum aAcc (59.8%) when the time window was 420 s. It can be seen that SIURUA can achieve better classification results than other algorithms when the time window = 300 s. It is worth noting that, although the time window is longer compared with DT, the best aAcc of SIURUA has a 16% improvement compared with the best aAcc of DT. When compared with SVM, NB, and LR, SIURUA improves the classification quality while shortening the time window required to obtain the best aAcc. Therefore, the suitable value of the time window for the SIURUA algorithm is chosen to be 300 s (5 min), and in our hybrid scene data, there are 146 keystroke operations and 314 mouse operations on average in 300 s.

It can be seen from Figure 8 that the authentication accuracy fluctuates. The authentication accuracy of SIURUA decreases slightly after 300 s, the authentication accuracy of DT decreases after 180 s, and the authentication accuracy of LR and SVM decreases after 360 s. The time window becomes longer, and the user’s operation within a time window changes. For example, in the Taobao scene, the user changes from browsing the product details page to browsing the product list page. The uncertainty brought by the switching of applications in the same scene affects the accuracy of the model authentication, and different models have different abilities to resist such interference.

As shown in Table 5, we compare the computational cost of SVM, DT, NB, LR, and SIURUA. The comparison is divided into two parts: building and authentication. The time complexity of building a classifier is decided by the machine learning algorithm [20]. For example, if we use SVM, the time complexity is $O\left(n^{3}\right)$, where n is the number of training data. The building time complexity using DT, NB, LR, and SIURUA is O(nmd), O(nmc), O(nm), and $O\left(m+n^{3}\right)$, where n is the number of training data, m is the feature dimension, d is the depth of decision tree, and c is the number of categories.

The authentication time of each algorithm is very rapid. Similar to the building process, the authentication time complexity using SVM, DT, NB, LR, and SIURUA is O(m), O(d), O(mc), O(m), and O(sm), respectively, where s is the number of support vectors.

#### 4.2.4. Determine Appropriate Kernel

The proposed SIURUA algorithm is based on EasyMKL, and the kernel of the EasyMKL algorithm is to use different kernel functions and weights to fuse data. The kernel functions need to be specified in advance, and the weights are obtained in the learning process. Therefore, this experience is to decide the appropriate kernels that are used to map scene-irrelated features and user-related features. As we know, linear kernels and RBF kernels have four combinations:

- Linear plus Linear (expressed as linear_linear) use linear kernels to map user-related features and linear kernels to map scene-irrelated features;
- Linear plus RBF (expressed as linear_rbf) use linear kernels to map user-related features and RBF kernels to map scene-irrelated features;
- RBF plus Linear (expressed as rbf_linear) use RBF kernels to map user-related features and Linear kernels to map scene-irrelated features;
- RBF plus RBF (expressed as rbf_rbf) use RBF kernels to map user-related features and RBF kernels to map scene-irrelated features.

**Figure 9:**
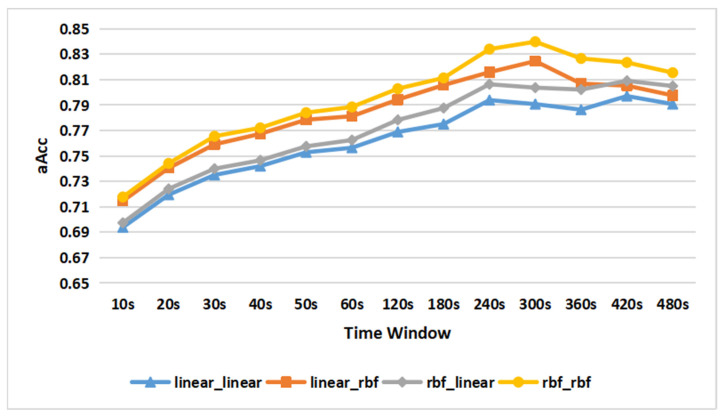
Comparison of the accuracy of SIURUA with different kernel functions.

This experiment selects the optimal kernel combination from the above four kernel combinations. The aAccs of the four kernel combinations with each value of the time window are shown in Figure 9, the number of feature combinations is 200 plus 200, and the time window = 300 s. It can be seen that in the case of RBF plus RBF and Linear plus RBF, the highest aAccs are 84% and 82.5% obtained in 300 s. In the case of Linear plus Linear and RBF plus Linear, the highest aAccs are 79.7% and 80.9% obtained in 420 s. We can see that RBF plus RBF gains the maximum aAcc in the smallest value of the time window. Therefore, the appropriate kernel combination for SIURUA is RBF plus RBF when the time window = 300 s and the number of feature combinations is 200 plus 200.

#### 4.2.5. Verify Feasibility of SIURUA

**Figure 10:**
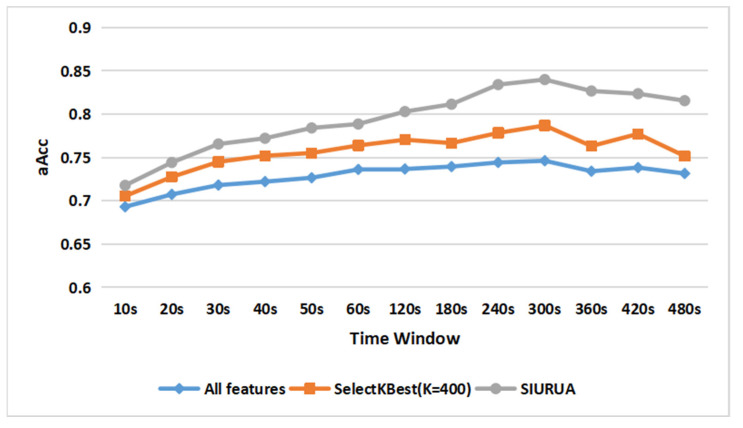
Comparison of accuracy between SIURUA and selecting 400 best features in a hybrid scene.

In order to further illustrate the feasibility of this algorithm, we use the features without feature selection and the features selected by mutual information as baselines. The number of features selected by SIURUA is 200 plus 200; in order to ensure the balance of the features, Mutual information needs to select the 400 features that have the highest correlation with users. Figure 10 shows the classification accuracy of different features with different values of the time window in hybrid scenes, where “All features” represents using whole feature sets to classify, SelectKBest (K = 400) indicates selecting 400 user-related features, and SVM with RBF kernel was selected as the classifier. We can see that the classification accuracy (75.2%) without feature selection is the worst. The classification accuracy (78.7%) with 400 user-related features has improved, and the SIURUA classification accuracy (84.0%) in the case of 200 plus 200 is the best. The 200 plus 200 features improve the accuracy of the model when using the same number of features as SelectKBest (K = 400), and this result proves that we successfully reduce the impacts of scene information on user-related features through fusing scene-irrelated features. Therefore, the result proves the feasibility of SIURUA.

**Figure 11:**
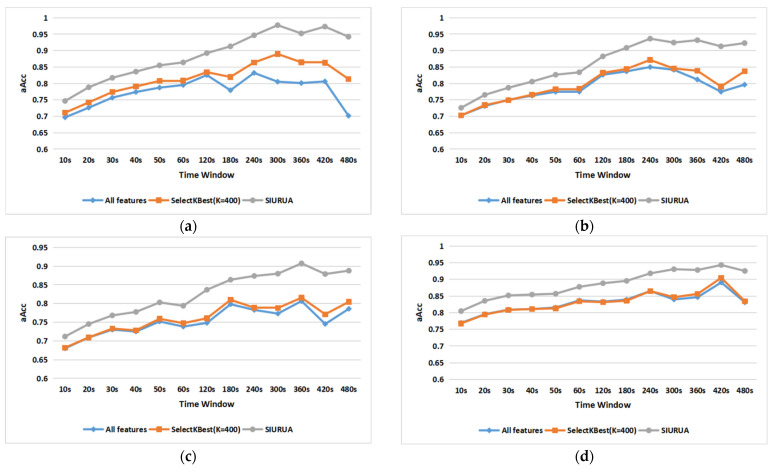
(**a**) Comparison of accuracy between SIURUA and the selection of 400 best features in a typing scene; (**b**) comparison of accuracy between SIURUA and the selection of 400 best features in a Taobao scene; (**c**) comparison of accuracy between SIURUA and the selection of 400 best features in a Weibo scene; (**d**) comparison of accuracy between SIURUA and the selection of 400 best features in a gaming scene.

For further proof, we employed this algorithm on four sets of single scene features, and the results are shown in Figure 11a–d. Figure 11a represents a typing scene, Figure 11b shows a Taobao scene, a Weibo scene is illustrated in Figure 11c, and a gaming scene is represented in Figure 11d. We can see that SIURUA can also improve the classification accuracy in the four single scenes. Although single-scene user authentication cannot be applied to the biometric system, the increasing accuracy can further verify the feasibility and versatility of SIURUA.

Combining the results of Figure 10 and Figure 11a–d, we can prove that fusing scene-irrelated features and user-related features can greatly improve the accuracy of the model, with the results verifying the feasibility of SIURUA.

#### 4.2.6. Determine Fill Values for Empty Features

**Figure 12:**
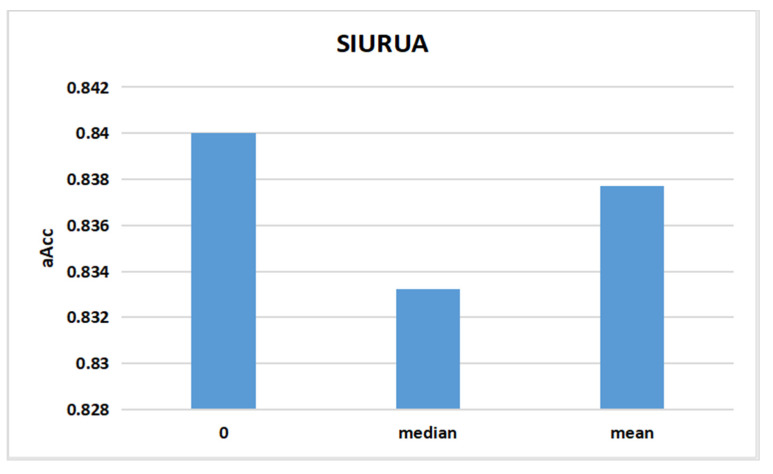
Comparison of accuracy between different fill values for empty features.

After determining all the parameters, we tested the time window = 300 s, the kernel combination rbf_rbf, and the feature combination 200_200 by selecting zero, the median, and the mean value to fill the empty features, as shown in Table 1. The experimental results are illustrated in Figure 12, which shows that the user authentication accuracy is highest for filling the empty features with zero and the lowest for filling with the median values. Therefore, we chose to fill the empty features in Table 1 with 0.

#### 4.2.7. Proven Advantages of SIURUA

This section will compare SIURUA with some proposed keystroke dynamics and mouse dynamics algorithms. The selected algorithms are as follows:

- MPCA [43]: Piantari et al. proposed a mouse dynamics user authentication method based on Principal Components Analysis (PCA) and SVM;
- UIKDMM [44]: Panasiuk et al. proposed a multimodal biometric user authentication system based on keystroke dynamics and mouse movements, which authenticates users through K-Nearest Neighbor (KNN) and by fusing keystroke dynamics and mouse dynamics;
- UAMKL [45]: Wang et al. proposed UAMKL, which is an AverageMKL-based keystroke dynamics and mouse dynamics fusion user authentication method;
- TEM [46]: Chen et al. proposed a multimodal biometric user authentication system based on keystroke dynamics and mouse dynamics with Context Information. The user authentication model in the system is a comparison, which fuses the SVM based on keystroke dynamics features and the NB based on mouse dynamics features by using the majority voting mechanism.

**Figure 13:**
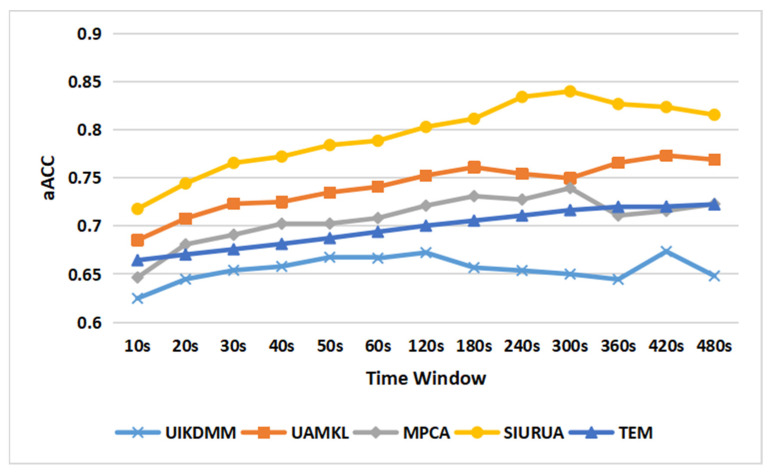
Comparison of accuracy between SIURUA and the proposed user authentication method.

Due to the particularity of a hybrid scene user keystroke dynamics and mouse dynamics dataset, we reproduced the above algorithms and experimented on multiple time window values. Figure 13 shows the authentication accuracy of SIURUA and the above four algorithms. It can be seen that the accuracy of SIURUA with all-time window values is better than that of some of the existing methods. The maximum aAcc of SIURUA is 84.0% at 300 s. UIKDMM and UAMKL achieve the maximum aAcc, 67.4%, and 77.3%, respectively, at 420 s. MPCA achieves the maximum aAcc 73.9% at 300 s. TEM achieves the maximum aAcc 72.2% at 480 s. Therefore, it is necessary to reduce the impacts of scene information in hybrid scenes, and SIURUA is superior to some of the existing algorithms.

#### 4.2.8. Comprehensive Comparison of Above Experiments

This section will summarize the various algorithms mentioned in Section 4.2.2, Section 4.2.3, Section 4.2.4, Section 4.2.5, Section 4.2.6 and Section 4.2.7. According to Section 4.2.2, Section 4.2.3, Section 4.2.4, Section 4.2.5, Section 4.2.6 and Section 4.2.7, when the time window = 300 s, the feature selection number is 200 plus 200, and the kernel combination is RBF plus RBF, SIURUA achieves the best accuracy, which is 84.0%. As shown in Table 6, it can be seen that SIURUA has obtained the highest aAcc, aPrec, aTPR, and aF1, as well as the lowest aFPR (aFAR) and aFRR of all the methods. Table 6 shows that SIURUA, as proposed in this paper, has excellent performance. It can obtain 0.840 aAcc, 0.841 aPrec, 0.85 aTPR, 0.169 aFPR (aFAR), 0.15 aFRR, and 0.839 aF1 with the conditions of time window = 300 s, 200 plus 200 features, and an RBF plus RBF kernel combination.

## 5. Conclusions and Future work

We summarize our results and discuss future research directions in the following sections.

### 5.1. Summaries and Discussion

The purpose of biometric technology based on keystroke dynamics and mouse dynamics is to simulate users’ behaviors and to find the distinguishing factors determining users’ identities. This paper not only fuses keystroke dynamics features and mouse dynamics features but also proposes a method to select the scene-irrelated features and user-related features for the fusion experiment in hybrid scenes for the first time. In the experiments, we tested the SIURUA algorithm on the collected hybrid scene data to verify the necessity of fusing scene-irrelated features and considered the possibility of SIURUA as a means of user authentication. Through the elaboration of SIURUA and the results of a comparison with other existing algorithms, it was found that the authentication performance of SIURUA in hybrid scenes with noise is better than other user authentication algorithms based on keystroke dynamics and mouse dynamics.

These results are encouraging and indicate that the proposed hybrid scene feature selection method and fusing the selected scene-irrelated features and user-related features can effectively improve the performance of the user authentication system.

### 5.2. Future Work

Although this paper verifies the feasibility of user authentication with hybrid scene features, we only considered four known scenes without verifying the application effect in more scenes or even unknown scenes. In the future, more hybrid scene data needs to be collected to further restrain the impact of scene information on the data. On the other hand, we do not consider the correlation between the scenes of the hybrid scene used in this paper. In addition, the feature selection method of hybrid scenes can be extended to be used not only in user authentication but also in other applications.

## Figures and Tables

**Table 1:** Examples of keystroke mouse dynamics features.

Feature	$f_{1}$	$f_{2}$	$f_{3}$	⋯	$f_{j}$
$KM-Feature_{1}$	$f_{1,1}$	$f_{1,2}$	$f_{1,3}$	⋯	$f_{1,j}$
$KM-Feature_{2}$	$f_{2,1}$	$f_{2,2}$	$f_{2,3}$	⋯	$f_{2,j}$
$KM-Feature_{3}$	$f_{3,1}$	$f_{3,2}$	$f_{3,3}$	⋯	$f_{3,j}$
⋮	⋮	⋮	⋮	⋱	⋮
$KM-Feature_{i}$	$f_{i,1}$	$f_{i,2}$	$f_{i,3}$	⋯	$f_{i,j}$

**Table 2:** Examples features of calculating AMI.

Feature	$Feature_{1}$	$Feature_{2}$	$Feature_{3}$	$Feature_{4}$	$Feature_{5}$
$f_{1}$	384	485	433	498	458
Scene	0	3	0	1	3

**Table 3:** Contingency table of $f_{j}$ and User.

$f_{j}\backslash User\;$	$U_{1}\;$	$U_{2}\;$	⋯	$U_{41}\;$	Sums
$F_{1,j}$	$nu_{1,1}$	$nu_{1,2}$	⋯	$nu_{1,41}$	$a_{1,j}$
$F_{2,j}$	$nu_{2,1}$	$nu_{2,2}$	⋯	$nu_{2,41}$	$a_{2,j}$
⋮	⋮	⋮	⋱	⋮	⋮
$F_{M_{j},j}$	$nu_{M_{j},1}$	$nu_{M_{j},2}$	⋯	$nu_{M_{j},41}$	$a_{M_{j},j}$
Sums	$b_{1}$	$b_{2}$	⋯	$b_{41}$	$\underset{kl}{\sum }nu_{kl}=N_{U}$

**Table 4:** Contingency table of $f_{j}$ and Scene.

$f_{j}\backslash Scene\;$	$S_{1}\;$	$S_{2}\;$	⋯	$S_{4}\;$	Sums
$F_{1,j}$	$ns_{1,1}$	$ns_{1,2}$	⋯	$ns_{1,41}$	$a_{1,j}$
$F_{2,j}$	$ns_{2,1}$	$ns_{2,2}$	⋯	$ns_{2,41}$	$a_{2,j}$
⋮	⋮	⋮	⋱	⋮	⋮
$F_{M_{j},j}$	$ns_{M_{j},1}$	$ns_{M_{j},2}$	⋯	$ns_{M_{j},41}$	$a_{M_{j},j}$
Sums	$c_{1}$	$c_{2}$	⋯	$c_{4}$	$\underset{kl}{\sum }ns_{kl}=N_{S}$

**Table 5:** Time complexity comparison of machine learning algorithms and SIURUA.

Algorithm	SVM	DT	NB	LR	SIURUA
Building	$O\left(n^{3}\right)$	O(nmd)	O(nmc)	O(nm)	$O\left(m+n^{3}\right)$
Authentication	O(m)	O(d)	O(mc)	O(m)	O(sm)

**Table 6:** Comprehensive comparison of the above methods.

Algorithm	aAcc	aPrec	aTPR	aFPR(aFAR)	aFRR
SVM	0.752	0.754	0.794	0.290	0.206
DT	0.712	0.714	0.716	0.291	0.284
NB	0.598	0.632	0.561	0.352	0.439
LR	0.758	0.742	0.790	0.282	0.210
SVM_400	0.766	0.762	0.798	0.266	0.202
MPCA [43]	0.739	0.735	0.770	0.290	0.230
UIKDMM [44]	0.674	0.674	0.705	0.335	0.295
UAMKL [45]	0.773	0.779	0.788	0.243	0.212
TEM [46]	0.722	0.713	0.782	0.333	0.217
SIURUA	**0.840**	**0.841**	**0.850**	**0.169**	**0.150**

## Data Availability

The data presented in this study are available on request from the corresponding author.
